# Acyclic Retinoid Attenuates STAT3 Signaling and Reduces In Vitro Growth of A375-Derived Dabrafenib Plus Trametinib-Resistant Melanoma Cells

**DOI:** 10.3390/ijms27146245

**Published:** 2026-07-14

**Authors:** Mitsuaki Nishizawa, Masanori Kimura, Hinata Hamada, Ichiro Yajima

**Affiliations:** Department of Bioscience and Engineering, College of Systems Engineering and Science, Shibaura Institute of Technology, Fukasaku 307, Minuma-ku, Saitama 337-8570, Japan

**Keywords:** melanoma, acyclic retinoid, peretinoin, dabrafenib, trametinib, drug resistance, STAT3, EGFR, Cyclin D1, p27KIP1

## Abstract

Resistance to combined BRAF and MEK inhibition remains a major barrier to durable disease control in BRAF-mutant melanoma. Acyclic retinoid (ACR; peretinoin) is a clinically studied retinoid, but its activity in MAPK inhibitor-resistant melanoma remains incompletely defined. Here, we established A375P-derived dabrafenib plus trametinib-resistant clones and evaluated ACR mainly in the A375PDTR-D clone as an in vitro proof-of-concept model. Resistant clones retained higher viability and failed to suppress ERK phosphorylation under dabrafenib plus trametinib treatment. In A375PDTR-D cells, ACR reduced short-term viability and clonogenic growth and was associated with decreased STAT3 Tyr705 phosphorylation, reduced EGFR, Cyclin D1, and Cyclin B1 expression, and increased p27KIP1. ACR did not detectably suppress AKT, MEK/ERK, or basal JNK phosphorylation under the tested conditions. These findings support further investigation of ACR as a candidate non-MAPK adjunct strategy; however, generalizability to broader melanoma models, formal drug-interaction status, direct cell-cycle/apoptosis effects, clinically achievable exposure, and in vivo efficacy remain to be established.

## 1. Introduction

Cutaneous melanoma remains a leading cause of cancer-related death despite advances in prevention and treatment. Globally, its incidence continues to rise, with over 325,000 new cases and approximately 57,000 deaths estimated in 2020, suggesting a public health burden that exceeds its proportion among all cancers [1,2]. Cutaneous melanoma acquires signaling plasticity under strong selective pressure, sustaining its proliferation, invasion, and metastasis.

Among these, the BRAF/MEK/ERK (MAPK), JAK/STAT, PI3K/AKT, and JNK/c-Jun pathways are clinically common and significant, supporting tumor growth, survival, and metastasis through a mutually complementary network of interactions among their components. First, BRAF/MEK/ERK (MAPK) is the primary driver of melanoma; MAPK signaling is hyperactivated by *BRAF* (V600E/K) activating mutations and *NRAS/NF1/KIT* abnormalities, promoting cell cycle entry, proliferation, and progression [3]. The JAK/STAT axis, particularly STAT3, is often constitutively activated in melanoma and contributes to its survival, motility, and metastasis. STAT3 is activated downstream of RTK and Src and is further induced by MAPK inhibition, supporting adaptive invasion [4].

The PI3K/AKT pathway is frequently dysregulated due to the loss or reduction in *PTEN* or upstream RTK activation, enhancing resistance to apoptosis, promoting proliferative and survival signaling, and interacting with MAPK output to contribute to malignancy and therapeutic resistance [5]. The JNK/c-Jun pathway is also implicated in melanoma malignancy, and JNK/c-Jun activation is associated with increased proliferation and shorter disease-free intervals in patients with melanoma. Pharmacological JNK inhibition has been reported to reduce c-Jun phosphorylation, suppress cell migration, and restore sensitivity to BRAF inhibition [6]. Collectively, these pathways function in a complementary and sometimes compensatory manner to drive cell cycle progression, survival signaling, epithelial–mesenchymal transition (EMT)-like plasticity, invasion, and organ-specific metastasis in cancer cells.

From a molecular biology perspective, *BRAF* activating mutations (mainly V600E, followed by V600K) are observed in approximately 40–50% of cutaneous melanomas and determine both tumor biology and therapeutic strategies [7,8,9,10]. BRAF and MEK inhibitors, particularly in combination therapy (e.g., dabrafenib plus trametinib), have surpassed previous standard treatments in both metastatic and postoperative adjuvant settings, significantly improving response rates, progression-free survival (PFS), and overall survival (OS) [11,12,13,14]. However, it has been reported that resistance to these anticancer agents is almost inevitable, often emerging within a few months and limiting the duration of therapeutic effectiveness [12,13,15,16,17]. This suggests the need for therapeutic strategies targeting non-MAPK survival pathways that can complement MAPK-targeted therapies.

Resistance mechanisms to BRAF/MEK inhibition are diverse, with MAPK reactivation being the most prevalent. Other contributing factors include *NRAS* mutations, *MEK1/2* mutations, *BRAF* amplification or splice variants, and pathway rewiring [15,16,17]. Additionally, receptor tyrosine kinase (RTK) signaling and PI3K-AKT activation can support cell survival via bypass pathways, with PDGFRβ, IGF1R, and, in some cases, the epidermal growth factor receptor (EGFR)/HER family involved in adaptive/acquired resistance [15,16,17,18,19,20]. Furthermore, changes in cell state, transcriptional reprogramming (e.g., *MITF-AXL* balance), transmission of resistance factors via extracellular vesicles, and the tumor microenvironment have been reported to enhance the resilience of melanoma to therapeutic pressure [16,18,19,20]. In other words, even under dual MAPK blockade by BRAF/MEK inhibitors, tumors can maintain proliferation and survival through non-MAPK pathways, indicating potential opportunities for further therapeutic intervention.

Clinically, immune checkpoint inhibitors (anti-PD-1/anti-CTLA-4) have established durable disease control in many patients with advanced melanoma [21,22], and recent treatment-sequencing studies support immunotherapy-first strategies for many patients with BRAF V600-mutant metastatic disease [23,24]. Nevertheless, BRAF/MEK-targeted therapy remains essential in selected adjuvant settings, in patients requiring rapid disease control, and after immunotherapy failure. Therefore, combination or sequential concepts with agents acting on non-MAPK survival pathways remain relevant for delaying resistance and extending disease control.

Retinoids regulate proliferation, differentiation, and apoptosis in a multifaceted manner through RAR/RXR-dependent transcriptional control and RAR-independent mechanisms. Among them, acyclic retinoids (ACR; peretinoin) have been the most extensively studied in clinical settings for preventing the recurrence of hepatocellular carcinoma (HCC), with trial results suggesting the suppression of secondary cancers after curative treatment [25,26,27,28,29]. Mechanistically, studies on various tumor types have reported that ACR and related retinoids regulate cell cycle/survival checkpoints by reducing EGFR Cyclin D1 levels, increasing cell cycle inhibitors such as p27^KIP1^, and suppressing STAT3 activity [30,31,32,33,34,35]. In liver cancer and squamous cell carcinoma models, ACR decreased p-EGFR, p-STAT3, and ERK levels, suppressed Cyclin D1 transcription, and promoted retinoid-responsive gene groups [30,32,35]. Although these findings are disease-dependent, they support the repositioning of ACR beyond HCC as a small molecule that inhibits proliferative signaling and G1/S control at multiple nodes with low-to-moderate toxicity.

In BRAF/MEK inhibitor-resistant melanoma, the ideal combination adjuvant agent should act downstream or in parallel to the MAPK pathway to avoid redundancy and induce cell cycle arrest. Retinoids suppress the survival and proliferation programs that persist after BRAF/MEK inhibition. Therefore, in this study, ACR (peretinoin) was investigated as a candidate adjuvant agent under conditions of resistance to dabrafenib plus trametinib. Using the human melanoma cell line A375P and a resistant strain established by long-term dual-inhibition exposure (A375PDTR), we tested the hypothesis that ACR exerts a complementary antitumor effect independent of MAPK pathway inhibition. Furthermore, it is known that activation of STAT3 promotes cell proliferation by enhancing the expression of Cyclin B1 and Cyclin D1, while suppressing the expression of p27^KIP1^. [36,37,38,39,40,41]. Specifically, it was hypothesized that ACR decreases the survival rate and colony-forming ability of resistant cells, induces rewiring characterized by reductions in EGFR, Cyclin B1, and Cyclin D1, increases in p27^KIP1^, and suppression of STAT3 phosphorylation, while having minimal influence on the phosphorylation of AKT, MEK/ERK, and JNK. This is consistent with the literature evidence that ACR regulates EGFR/STAT3 and cell cycle output rather than directly inhibiting MAPK [30,31,32,33,34,35]. If demonstrated, this would provide a rationale for using non-MAPK therapeutic levers to reduce the viability of dabrafenib plus trametinib-resistant melanoma cells.

In this study, we evaluated the effects of ACR on melanoma, particularly on BRAF/MEK inhibitor-resistant cancer cells, an area where insights into the multi-pathway regulation of ACR, as demonstrated in HCC and squamous cell carcinoma, are insufficient. Furthermore, we believe that low-toxicity small molecules that do not amplify MAPK pathway-related adverse events could be advantageous as part of long-term maintenance strategies for resistant cases of melanoma. Overall, we propose the potential utility of ACR as an agent that acts through a pathway distinct from MAPK-targeted therapies in BRAF V600-mutant melanoma. Considering the urgent epidemiological need and the benefits and limitations of BRAF/MEK inhibitor, EGFR/STAT3 inhibition, and cell cycle regulation, we aimed to verify that ACR can suppress survival and colony formation in dual inhibitor-resistant melanoma while having minimal effect on MAPK/AKT/JNK phosphorylation. This is directly linked to combination designs with dabrafenib plus trametinib, highlighting the potential of retinoid-based adjunct strategies to delay resistance and prolong disease control.

## 2. Results

### 2.1. ACR Further Reduces Viability Under Selected Dabrafenib and Trametinib Conditions in Parental A375P Cells

**Figure 1:**
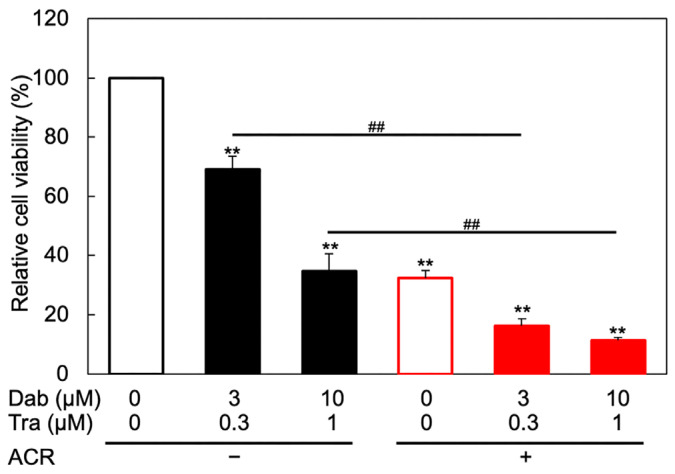
ACR enhances the antitumor effect of dabrafenib/trametinib in parental A375P cells. A375P cells were treated with dabrafenib (Dab) and trametinib (Tra), in combination with ACR (20 μM), for 72 h. Values are expressed as percentages normalized to the mean of the vehicle (DMSO) control. Bars indicate mean ± SD (*n* = 4). Statistical analysis was performed using one-way ANOVA, followed by the Tukey–Kramer multiple-comparison test. Asterisks indicate significant differences from the vehicle group (** *p* < 0.01). Sharps Indicates significant differences between the non-exposed and ACR-exposed to ACR at the same concentrations of Dab + Tra (## *p* < 0.01).

To evaluate the growth-suppressive effect of ACR under selected dabrafenib plus trametinib conditions in parental A375P melanoma cells harboring *BRAF*V600E, cells were exposed to dabrafenib, trametinib, and ACR for 72 h, and cell viability was measured (Figure 1). In the absence of ACR, dabrafenib plus trametinib treatment led to a concentration-dependent decrease in cell viability, indicating that A375P cells are sensitive to dual MAPK inhibition. When ACR (20 μM) was combined, cell viability was further reduced under several drug conditions compared with the corresponding conditions without ACR. These data indicate that ACR can further reduce viability under selected in vitro combination conditions; however, formal Bliss, Loewe, ZIP, HSA, or Chou-Talalay combination-index analyses were not performed, and the present data should not be interpreted as demonstrating synergy or additivity.

### 2.2. Establishment and Characterization of Dabrafenib Plus Trametinib-Resistant A375P Derivatives

**Figure 2:**
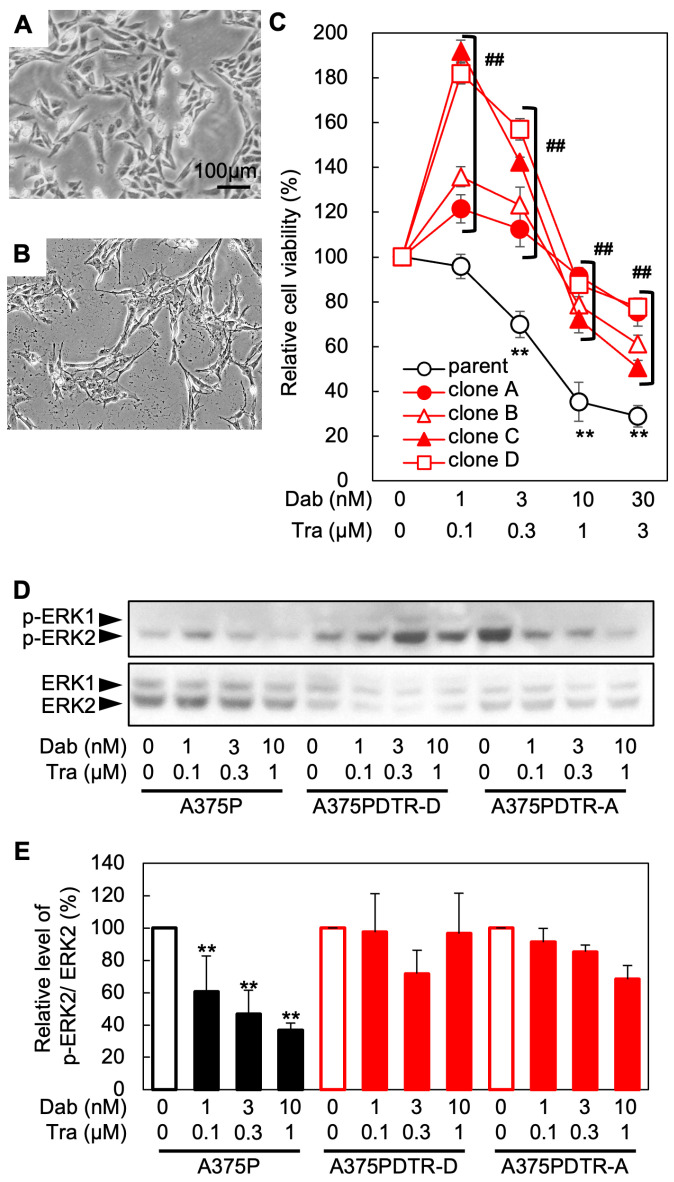
Establishment of resistant A375PDTR clones through long-term exposure to dabrafenib plus trametinib. The parental A375P cell line was exposed stepwise to dabrafenib (Dab) + trametinib (Tra) for 6 months, resulting in the establishment and evaluation of four resistant clones (A375PDTR-A–D). (**A**,**B**): Representative morphologies of A375P (**A**) and A375PDTR-D (**B**) cells. (**C**): Dose-dependent survival rate (72 h) upon combined Dab + Tra treatment in A375P and A375PDTR-A to D. Results were normalized to the average of the vehicle (DMSO) control and expressed as percentages. Bars indicate mean ± SD (*n* = 4). Statistical analysis was performed using one-way ANOVA, followed by the Tukey–Kramer multiple comparison test. Asterisks indicate statistically significant differences compared with the vehicle (DMSO) control in parental A375P cells (** *p* < 0.01). Sharps indicate significant differences compared with parental A375P cells under the same dabrafenib plus trametinib condition (## *p* < 0.01). (**D**): Changes in ERK1/2 phosphorylation levels upon exposure to Dab + Tra in each cell line. (**E**): The relative amounts of p-ERK2/ERK2 were quantified and normalized to the vehicle = 100% for each cell line. Bars indicate mean ± SD (*n* = 4). Statistical analysis was performed using one-way ANOVA (Tukey–Kramer), with statistical significance compared to the vehicle control within the same cell line, indicated by (** *p* < 0.01).

A375P cells were exposed to a combination of dabrafenib and trametinib for six months, and four single-cell-derived resistant clones (A375PDTR-A–D) were generated. Figure 2A,B show the representative morphology of the parental strain and one resistant clone (A375PDTR-D). The drug responses of parental A375P and four A375PDTR clones to dabrafenib and trametinib were compared. Under combination treatment, each resistant clone consistently showed higher survival rates than the parental strain under the same drug conditions (Figure 2C). These results indicate that each clone acquired drug resistance. To examine the changes in intracellular signaling pathways associated with drug resistance, we measured ERK activity. In parental A375P cells, combination treatment with dabrafenib and trametinib significantly decreased p-ERK1/2 levels, whereas no such decrease was observed in resistant clones (Figure 2D,E). These results demonstrate that even under dual BRAF/MEK inhibitor treatment, resistant clones are characterized as drug-resistant clones. To confirm the generality of these findings, the same analyses were performed on A375PDTR-B and A375PDTR-C cells. In these independent clones, no significant decrease in p-ERK1/2 was observed following combination treatment with dabrafenib and trametinib (Supplementary Figure S1), supporting consistent ERK suppression failure across clones.

### 2.3. ACR Reduced the Short-Term Viability and Long-Term Clonogenic Survival of the Resistant Clone A375PDTR-D

**Figure 3:**
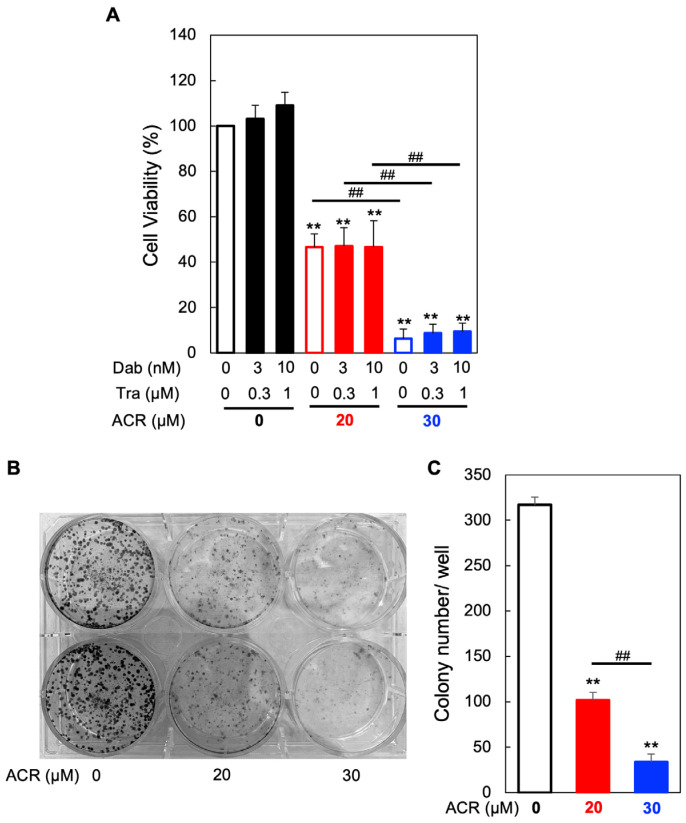
ACR significantly suppressed the survival rate and colony-forming ability of dabrafenib plus trametinib-resistant melanoma cells. (**A**): Relative survival rate of A375PDTR-D cells after 72 h of treatment with dabrafenib (Dab), trametinib (Tra), and ACR. Values were normalized to the mean of the vehicle (DMSO) control and expressed as a percentage. Bars represent the mean ± SD (*n* = 5). Statistical analysis was performed using one-way ANOVA followed by the Tukey–Kramer multiple comparison test. The asterisks indicate significant differences compared to the vehicle (DMSO) control (** *p* < 0.01). Sharps in the figure indicate significant differences between 20 μM and 30 μM ACR at the same Dab + Tra concentration (## *p* < 0.01). (**B**): Representative image of the colony formation assay of A375PDTR-D cells. (**C**): Quantification of colony numbers. Values were normalized to the mean of the vehicle (DMSO) control and expressed as a percentage. Bars represent the mean ± SD (*n* = 4). Statistical analysis was performed using one-way ANOVA followed by the Tukey–Kramer multiple comparison test. Asterisks indicate significant differences compared to the vehicle (DMSO) control (** *p* < 0.01). Sharps in the figure indicate significant differences between ACR concentrations (## *p* < 0.01).

To evaluate the antitumor effect of ACR on melanoma cells after acquiring drug resistance, A375PDTR-D cells were treated with dabrafenib, trametinib, and ACR, and their viability was measured. Under multiple dabrafenib–trametinib exposure conditions, combined treatment with ACR further reduced cell viability compared to conditions without ACR (Figure 3A). Next, the clonogenic ability of drug-resistant melanoma cells was assessed using a colony assay. Visible decreases in both colony numbers and sizes were observed after ACR treatment. Quantitative analysis further showed that colony numbers were significantly reduced by ACR at 20 and 30 μM (Figure 3C). These results indicate that ACR suppresses both short-term viability and long-term clonogenic ability in dabrafenib plus trametinib-resistant melanoma cells, suggesting its potential as a novel drug candidate for resistant melanomas.

### 2.4. ACR Suppresses STAT3 Activation in the Resistant Clone A375PDTR-D

**Figure 4:**
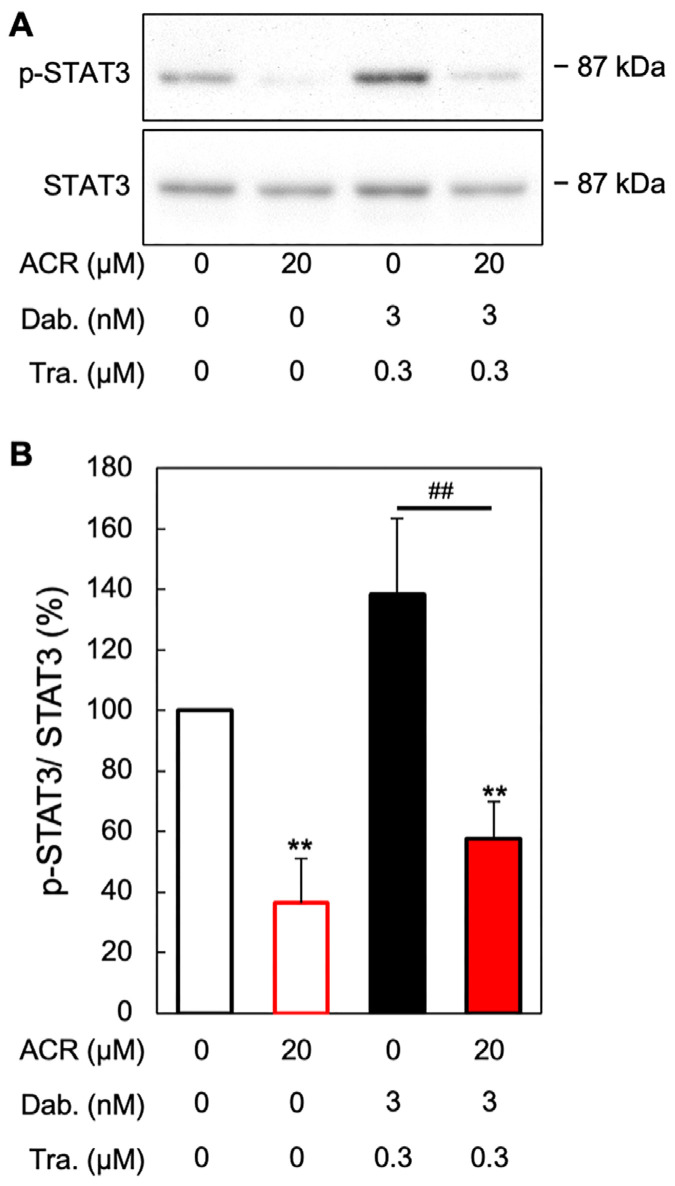
ACR reduces STAT3 phosphorylation (Tyr705) in dabrafenib plus trametinib-resistant melanoma cells. (**A**): Western blot analysis of phospho-STAT3 (Tyr705) and total STAT3 in A375PDTR-D cells. (**B**): Changes in phospho-STAT3 levels following ACR treatment in dabrafenib plus trametinib-resistant cells. Values are expressed as percentages normalized to the mean of the vehicle (DMSO) control. The bars represent the mean ± SD (*n* = 4). Statistical analysis was performed using one-way ANOVA followed by the Tukey–Kramer multiple comparison test. Asterisks indicate significant differences compared to the vehicle (DMSO) control (** *p* < 0.01). Sharps indicate significant differences depending on the presence or absence of ACR at the same concentration of Dab + Tra (## *p* < 0.01).

The JAK/STAT axis, particularly STAT3, is often constitutively activated in melanoma and contributes to cell survival, motility, and metastasis. STAT3 is activated downstream of RTKs and Src and is further induced by MAPK inhibition to support adaptive invasion [4]. To determine whether ACR treatment was associated with changes in STAT3 activation, we examined phospho-STAT3 (Tyr705) levels in A375PDTR-D cells. In the absence of ACR, no clear changes were observed in phospho-STAT3 (Tyr705) levels. In the presence of ACR, phospho-STAT3 (Tyr705) levels were reduced regardless of the presence or absence of dabrafenib and trametinib (Figure 4). These results indicate that, under the tested conditions, ACR treatment was associated with reduced STAT3 Tyr705 phosphorylation.

### 2.5. ACR Did Not Detectably Suppress AKT, MEK/ERK, or Basal JNK Phosphorylation in A375PDTR-D Cells

**Figure 5:**
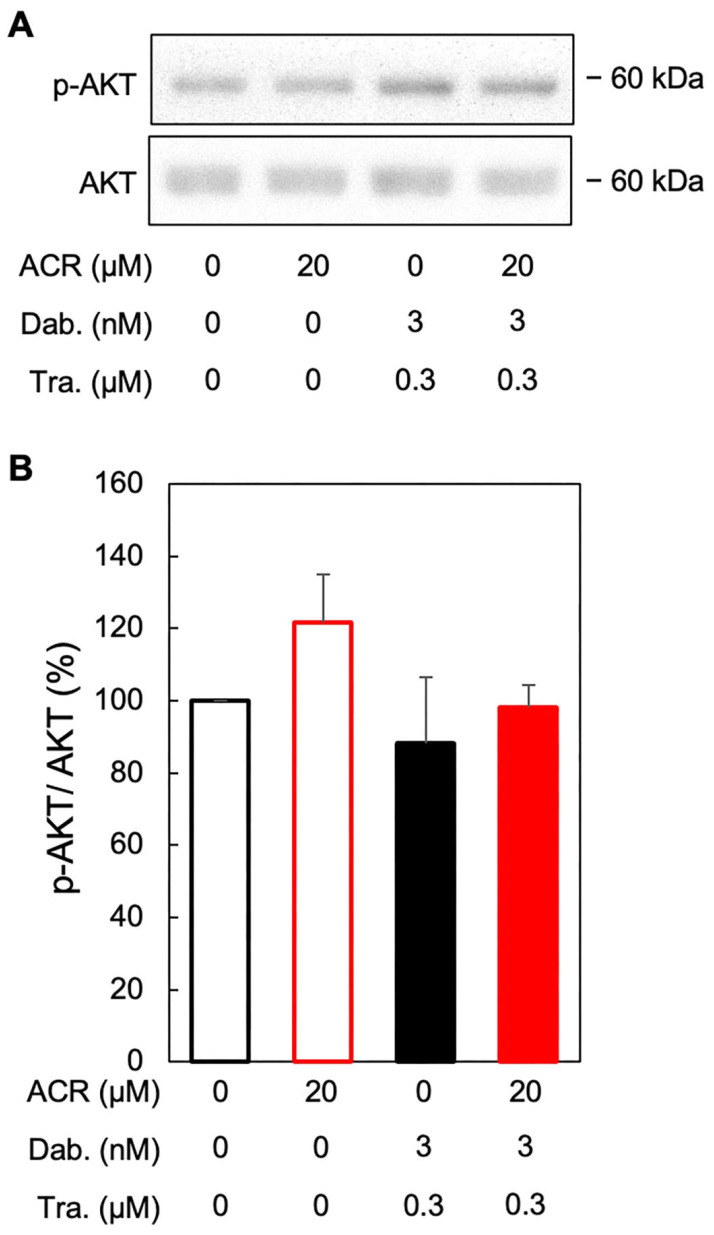
ACR did not detectably suppress AKT phosphorylation in dabrafenib plus trametinib-resistant melanoma cells under the tested conditions. (**A**): Western blot analysis of phospho-AKT and total AKT in A375PDTR-D cells. (**B**): Changes in phospho-AKT levels following ACR treatment in dabrafenib plus trametinib-resistant cells. Values are presented as percentages normalized to the mean of the vehicle (DMSO) control. Bars indicate the mean ± SD (*n* = 3). No significant difference was observed versus vehicle (one-way ANOVA followed by Tukey–Kramer).

The PI3K/AKT pathway is frequently disrupted by upstream changes in *PTEN* or RTK activity, which not only enhance resistance to apoptosis and promote growth and survival signals, but also interact with MAPK signaling to contribute to drug resistance [5]. To determine whether the antitumor effect of ACR involves the PI3K/AKT pathway, we examined the changes in phospho-AKT levels in A375PDTR-D cells. Consequently, ACR treatment did not affect phospho-AKT levels, regardless of the presence or absence of dabrafenib plus trametinib (Figure 5).

**Figure 6:**
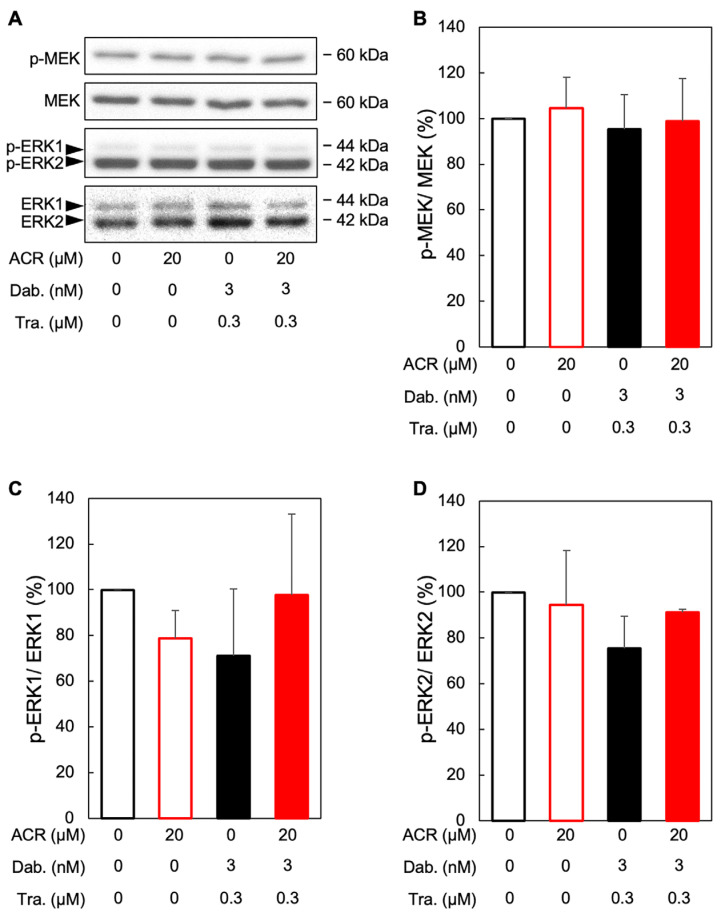
ACR did not detectably suppress MEK/ERK phosphorylation in dabrafenib plus trametinib-resistant melanoma cells under the tested conditions. (**A**): Western blot analysis of p-MEK, total MEK, p-ERK1/2, and total ERK1/2 in A375PDTR-D cells. (**B**–**D**): Changes in p-MEK and p-ERK2 levels following ACR treatment of dabrafenib plus trametinib-resistant cells. Values were normalized to the mean of the vehicle (DMSO) control and are displayed as percentages. Bars indicate mean ± SD (*n* = 3). No significant difference was observed versus vehicle (one-way ANOVA followed by Tukey–Kramer).

The BRAF/MEK/ERK (MAPK) pathway is one of the most highly activated pathways in melanoma, and MAPK signaling is excessively activated by *BRAF* (V600E/K) activating mutations or *NRAS/NF1/KIT* abnormalities, thereby promoting proliferation and invasion [3]. To determine whether ACR treatment was accompanied by detectable changes in the MAPK pathway, we examined the changes in phospho-MEK and ERK levels in A375PDTR-D cells. Under the tested conditions, ACR treatment did not detectably suppress phospho-MEK or phospho-ERK levels, regardless of the presence or absence of dabrafenib and trametinib (Figure 6).

**Figure 7:**
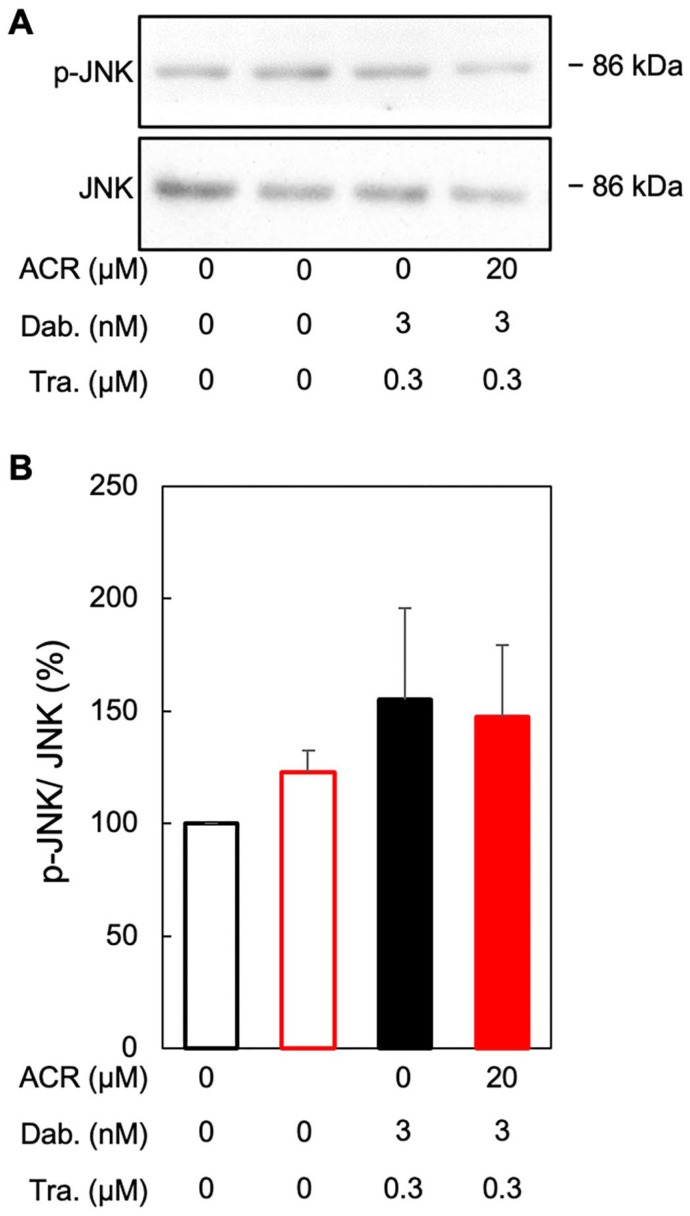
ACR did not detectably suppress basal JNK phosphorylation in dabrafenib plus trametinib-resistant melanoma cells under the tested conditions. (**A**): Western blot of phospho-JNK and total JNK in A375PDTR-D cells. (**B**): Changes in phospho-JNK levels in dabrafenib plus trametinib-resistant cells after ACR treatment. Values are expressed as a percentage normalized to the mean of the vehicle (DMSO) control. Bars represent the mean ± SD (*n* = 3). No significant difference was observed versus vehicle (one-way ANOVA followed by Tukey–Kramer).

The JNK/c-Jun pathway is involved in melanoma malignancy, and JNK/c-Jun activation has been associated with increased cell proliferation and shortened relapse-free periods [6]. To clarify whether ACR treatment was accompanied by detectable changes in basal JNK/c-Jun signaling, we examined the changes in phospho-JNK levels in A375PDTR-D cells treated with ACR. Under the tested conditions, ACR treatment did not detectably suppress phospho-JNK levels, regardless of the presence or absence of dabrafenib plus trametinib (Figure 7).

These findings indicate that, under the tested conditions, ACR is associated with STAT3 attenuation and changes in EGFR/cell-cycle regulatory proteins, whereas AKT, MEK/ERK, and basal JNK phosphorylation were not detectably suppressed.

### 2.6. ACR Modulates EGFR and Cell-Cycle Regulators in A375PDTR-D Cells

**Figure 8:**
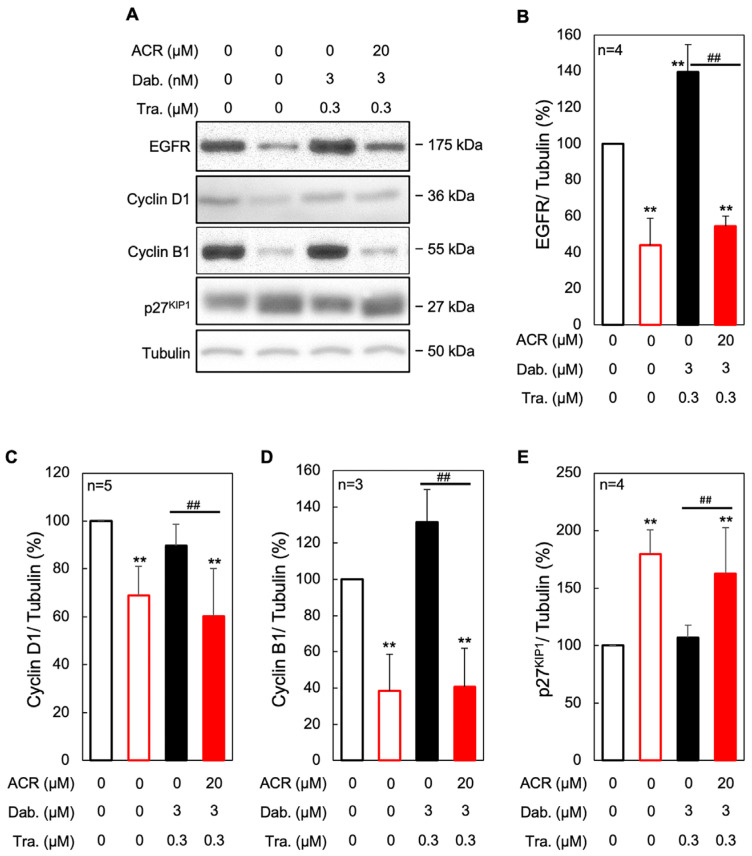
In dabrafenib plus trametinib-resistant melanoma cells, the levels of EGFR, Cyclin D1 and Cyclin B1 were reduced, whereas the level of p27^KIP1^ was increased. (**A**): Western blot analysis of EGFR, Cyclin D1, Cyclin B1 and p27^KIP1^ in A375PDTR-D cells. Tubulin was detected as a loading control. (**B**–**E**): Densitometric quantification of EGFR (**B**), Cyclin D1 (**C**), Cyclin B1 (**D**) and p27^KIP1^ (**E**). Each signal was normalized to tubulin, then normalized to the mean value of the vehicle (DMSO) control, and expressed as a percentage. Bars represent the mean ± SD (*n* = 4). Statistical analysis was performed using one-way ANOVA, followed by Tukey–Kramer multiple comparisons. Asterisks indicate significant differences compared to the vehicle group (** *p* < 0.01). Sharps indicate a significant difference in the presence or absence of ACR at the same concentration of Dab + Tra (## *p* < 0.01).

It has been reported that ACR and related retinoids are involved in the expression and activity of EGFR, which is upstream of the JAK/STAT pathway, as well as Cyclin D1, Cyclin B1, and p27KIP1, which are related to cell-cycle control [30,31,32,33,34,35]. To assess whether ACR-associated growth reduction was accompanied by changes in EGFR and cell-cycle/JAK/STAT-related proteins, we examined changes in the expression of EGFR, Cyclin D1, Cyclin B1, and p27KIP1. Regardless of the presence or absence of dabrafenib plus trametinib, ACR treatment significantly decreased EGFR, Cyclin D1, and Cyclin B1 levels and significantly increased p27KIP1 levels (Figure 8). These results suggest that ACR treatment is associated with reduced EGFR expression and changes in JAK/STAT pathway-related proteins, which may contribute to reduced proliferation.

## 3. Discussion

**Figure 9:**
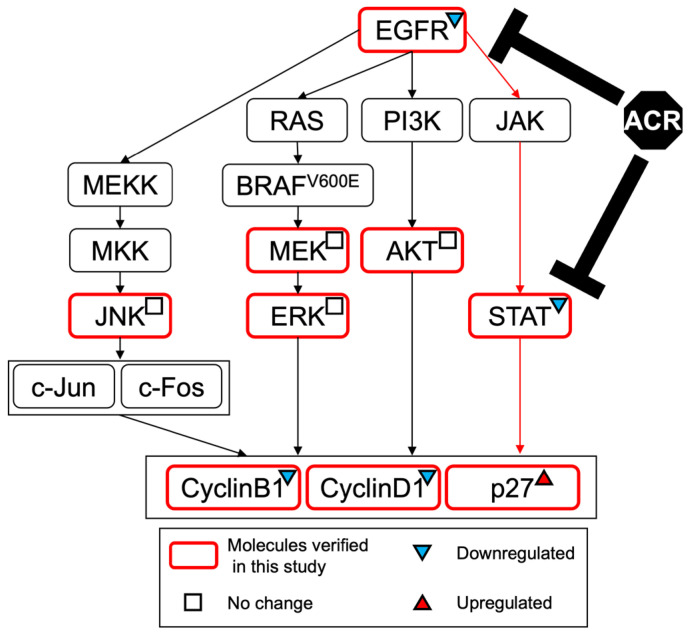
Proposed working model based on the associations observed in this study. Under BRAF/MEK inhibitor resistance (dabrafenib plus trametinib), ERK phosphorylation is not sufficiently suppressed (incomplete MAPK inhibition). ACR was associated with reduced STAT3 Tyr705 phosphorylation and changes in EGFR/cell-cycle regulators, while AKT, MEK/ERK, and basal JNK phosphorylation were not detectably suppressed under the tested conditions (Figure 5, Figure 6, Figure 7 and Figure 8). These molecular changes coincided with reduced short-term viability and clonogenic growth in A375PDTR-D cells (Figure 3). This model is hypothesis-generating and does not establish pathway causality.

In this study, four dabrafenib (D) + trametinib (T)-resistant clones established from A375P demonstrated higher survival rates than the parental line, even under dual MAPK inhibition, and failed to suppress ERK phosphorylation upon dabrafenib plus trametinib exposure, thereby confirming both functional and biochemical resistance. Under these conditions, ACR (peretinoin) reduced short-term viability and clonogenic growth of A375PDTR-D cells. Consistent with an association-based model, ACR treatment was accompanied by reduced STAT3 phosphorylation (Tyr705), decreased EGFR, Cyclin D1, and Cyclin B1 levels, and increased p27KIP1. These findings suggest changes in receptor-signaling and cell-cycle regulatory outputs, but they do not establish a causal pathway or direct G1/S arrest. In contrast, AKT, MEK/ERK, and basal JNK phosphorylation were not detectably suppressed under the tested conditions. Taken together (Figure 9), these findings support an association-based working model in which ACR reduces in vitro growth in A375-derived dabrafenib plus trametinib-resistant melanoma cells without demonstrating pathway causality or MAPK/AKT/JNK pathway independence.

Acquired resistance to BRAF/MEK inhibition is multifaceted, and several studies have described mechanisms such as NRAS/MEK gene mutations, BRAF gene amplification and splice variants, MAPK reactivation through downstream pathway rewiring, and survival bypass pathways involving RTKs and PI3K/AKT signaling [15,16,17,18,19,20,42,43,44]. In early mechanistic studies, Nazarian et al. presented increased PDGFRβ expression and *NRAS* mutations as mutually exclusive drivers of BRAF inhibitor resistance, highlighting both RTK bypass signaling and RAS-dependent pathway reactivation [15]. Subsequent integrative studies have demonstrated RTK-driven adaptive responses, including those involving the ERBB family, transcriptional state changes, and the influence of the tumor microenvironment [17,42,43,44]. The finding in this study that A375PDTR cells failed to show a decrease in p-ERK is consistent with this trend and supports the need to examine non-MAPK adjunct strategies.

Among RTKs, EGFR/HER-family signaling is relevant to resistance in BRAF-mutant melanoma. Sun et al. demonstrated that some BRAF-inhibitor-resistant melanoma cells acquire EGFR expression and that EGFR can enhance proliferation under BRAF/MEK inhibition [43]. Epigenetic upregulation of EGFR and overactivation of the EGFR/PI3K/AKT pathway have also been reported in BRAF inhibitor-resistant cutaneous melanoma [45], while *FOXD3*-mediated *ERBB3* upregulation provides another ligand-responsive escape route [42]. These studies support the broader concept that RTK/ERBB-family context can influence response to BRAF/MEK inhibition. In this study, the decrease in EGFR protein expression level by ACR (Figure 8) is consistent with classical findings that retinoid treatment can reduce EGFR-related signaling in other tumor models [32]. A recent exploratory biomarker analysis from the phase III COLUMBUS study further supports the clinical relevance of ERBB-family and tumor-context biomarkers in BRAF/MEK-targeted therapy [46]. In that analysis, clinical benefit from encorafenib plus binimetinib versus vemurafenib varied according to molecular features, including tumor ERBB2 expression context. Although our study examined EGFR rather than ERBB2 and used an in vitro A375-derived resistant model, these clinical transcriptomic data support the broader concept that RTK/ERBB-family context can influence response to BRAF/MEK inhibition and may identify bypass states relevant to resistance. Therefore, the COLUMBUS analysis is cited as clinical context for ERBB-family relevance in targeted therapy, not as direct evidence that ERBB2 was involved in the present model.

STAT3 integrates RTK/Src/JAK pathways and regulates transcriptional programs related to cell survival, invasion, and immune-context signaling. Vultur et al. demonstrated that MEK/BRAF inhibition can induce STAT3 signaling and invasive behavior in human melanoma models and that STAT3 inhibition was particularly effective in a 3D context [47]. The reduction in p-STAT3 by ACR (Figure 4) is also consistent with previous reports that ACR and related retinoids affect EGFR/STAT3- and cell-cycle-associated signaling in other tumor contexts [30,31,32,33,34,35]. In the present study, however, these observations should be interpreted as associations, because rescue or perturbation experiments were not performed to prove that STAT3 attenuation is required for ACR-mediated growth reduction.

Excessive Cyclin D1 levels and reduced p27KIP1 levels are typical drivers of G1 progression. Retinoids have classically been shown to affect Cyclin D1 turnover/expression and p27KIP1 regulation [30,31,32,33,34,35,48,49]. In this study, ACR treatment was associated with reduced Cyclin D1 and Cyclin B1 levels and increased p27KIP1 in resistant melanoma cells. These changes are consistent with reduced proliferative output, but direct cell-cycle distribution, DNA-synthesis, and apoptosis assays were not performed. Thus, the present data support an association-based model linking ACR treatment to altered EGFR/STAT3 and cell-cycle regulatory proteins, rather than proving direct G1/S or G2/M arrest.

ACR/retinoids have been reported to affect p-ERK levels and EGFR-related signaling in liver and other tumor systems [26,32,35]. However, in the drug-resistant melanoma system studied here, no significant changes were observed in p-AKT, p-MEK/ERK, or p-JNK levels. This finding should be interpreted cautiously because the AKT, MEK/ERK, and JNK immunoblot analyses were performed with *n* = 3, and the JNK assay lacked a pathway-activation positive control. Nevertheless, the observed changes in STAT3/EGFR and cell-cycle regulatory proteins suggest that downstream transcriptional and proliferative outputs may remain modifiable even when proximal MAPK signaling is not detectably suppressed.

Even as adjuvant therapy, dabrafenib plus trametinib prolongs recurrence-free survival, with sustained effects demonstrated at five years [12], and long-term outcomes of BRAF/MEK-targeted therapy in metastatic melanoma have also been reported [13]. Immune checkpoint inhibition has also improved long-term survival in advanced melanoma, with 10-year CheckMate-067 data reported for nivolumab plus ipilimumab [22]. Treatment-sequencing studies support immunotherapy-first approaches for many patients with BRAF-mutant metastatic melanoma, while targeted therapy remains clinically important in selected contexts [23,24]. Within this clinical ecosystem, our data suggest that ACR may modulate EGFR/STAT3 and cell-cycle regulatory outputs in vitro without detectable suppression of MAPK/AKT/JNK phosphorylation under the tested conditions. These properties support further preclinical evaluation of ACR as a candidate non-MAPK adjunct strategy, but formal drug-interaction analyses, clinically achievable exposure, tolerability in melanoma models, and in vivo efficacy remain to be established.

Peretinoin (ACR) has been clinically studied mainly in the hepatocellular carcinoma setting [27,50,51]. The consistent trends observed in this study, including decreased EGFR, p-STAT3, and Cyclin D1 levels and increased p27KIP1, are broadly consistent with previous retinoid-related studies. However, ACR was used at 20–30 μM in vitro in the present melanoma experiments; reported clinical plasma exposure after oral peretinoin dosing is lower than this range [50], and whether these concentrations are achievable in melanoma tissue after clinically used peretinoin dosing is unknown. Therefore, the pharmacological gap between the in vitro concentration range and clinically achievable exposure should be treated as a limitation rather than as evidence of direct translational feasibility.

Strategies to delay or overcome BRAF/MEK inhibitor resistance include vertical MAPK inhibition, RTK/Src or ERBB-family blockade, FAK/YAP-related interventions, and combination with immunotherapy [15,16,17,18,19,20,42,43,44,45,47,52,53,54], but each comes with challenges such as toxicity and cross-resistance. In this study, we focused on ACR, which was associated with changes in the EGFR-STAT3-cell-cycle regulatory axis, and showed its potential for further evaluation as a non-MAPK adjunct candidate with dabrafenib plus trametinib. These association-based features suggest that ACR may reduce proliferative output even when proximal signaling is buffered. Going forward, verification related to interaction analysis (Bliss/Loewe/ZIP/HSA) and timing sequences (simultaneous/sequential) will be necessary.

This study has several important limitations. First, the ACR-response experiments were performed mainly in one A375-derived resistant clone, A375PDTR-D. Although four resistant clones were generated and additional clones supported failure of ERK suppression under dabrafenib plus trametinib, the effects of ACR on viability, colony formation, STAT3 phosphorylation, EGFR, and cell-cycle regulators were not validated across multiple independent clones or patient-derived melanoma models. Therefore, the findings should be interpreted as in vitro proof-of-concept data rather than as evidence of a broadly generalizable effect in resistant melanoma. Second, the molecular basis of resistance in A375PDTR clones was not defined by genomic or transcriptomic profiling, and resistance mechanisms such as *BRAF* amplification or splice variants, NRAS/MEK alterations, RTK rewiring, and *MITF/AXL* state changes remain unresolved. Third, the observed decreases in p-STAT3, EGFR, Cyclin D1, and Cyclin B1 and the increase in p27KIP1 are associative; rescue, knockdown, or pharmacological comparator studies were not performed to establish that EGFR or STAT3 suppression is required for ACR-mediated growth reduction. Fourth, formal drug-interaction analyses were not performed, so the effects of ACR with dabrafenib plus trametinib cannot be classified as synergistic or additive. Fifth, direct assays of cell-cycle distribution, DNA synthesis, apoptosis, or cell death were not performed, and metabolic viability and colony assays cannot distinguish cytostatic effects from apoptosis or metabolic suppression. Sixth, all experiments were performed in vitro, without animal efficacy, tolerability, or immune-context evaluation. Seventh, the *n* = 3 immunoblot analyses for AKT, MEK/ERK, and JNK may be underpowered to exclude modest pathway changes, and the JNK immunoblotting did not include a pathway-activation positive control; therefore, nonsignificant results should not be interpreted as definitive evidence of pathway independence. Finally, ACR was used at 20–30 μM in vitro; the relationship between these concentrations and clinically achievable peretinoin exposure remains uncertain. These issues define the next experimental steps required before clinical translation can be inferred.

## 4. Materials and Methods

### 4.1. Cell Lines, Authentication, and General Culture

Cell culture: The human melanoma cell line A375P was supplied by the American Type Culture Collection (Manassas, VA, USA) and cultured in RPMI-1640 medium supplemented with 10% fetal bovine serum and 1% penicillin/streptomycin (FUJIFILM Wako Chemicals, Osaka, Japan) at 37 °C in 5% CO_2_. The cells were routinely subcultured every 2–3 days to maintain exponential growth. Mycoplasma contamination was tested using a MycoAler Mycoplasma Detection Kit (Lonza, Basel, Switzerland) and was found to be negative for mycoplasma. For the experimental assays, cells were seeded at appropriate densities to ensure logarithmic growth during the treatment period.

### 4.2. Establishment of Dabrafenib Plus Trametinib-Resistant Clones

A375P cells were cultured for six months while being exposed to gradually increasing concentrations of dabrafenib and trametinib. The selection protocol started at 100 nM dabrafenib plus 1 nM trametinib. The dabrafenib concentration was increased by approximately 10 nM at approximately 1-week intervals until reaching 250 nM, while trametinib was co-administered and ultimately maintained at 10 nM. Cells were maintained continuously in drug-containing medium, with medium replacement every 3 days, and passaged from approximately 10–20% to 80–90% confluence. After six months of exposure, four single-cell-derived clones (A–D) were isolated and expanded. Unless otherwise specified, clone D (A375PDTR-D) was used in the main figures, whereas clones B and C were used in Supplementary Figure S1 to assess whether ERK suppression failure was also observed in additional resistant clones.

### 4.3. Reagents and Treatments

Acyclic retinoids were purchased from Sigma-Aldrich (St. Louis, MO, USA), and dabrafenib and trametinib were purchased from Funakoshi (Tokyo, Japan). Rabbit polyclonal antibodies against ERK1/2, phospho-STAT3 (Tyr705), STAT3, Epidermal growth factor receptor (EGFR), phospho-AKT, p27^KIP1^, phospho-JNK1/2 and JNK1/2 were purchased from Cell Signaling Technology (Danvers, MA, USA). Mouse monoclonal antibodies against phospho-ERK1/2, Cyclin B1 and alpha-tubulin were purchased from Sigma-Aldrich (St. Louis, MO, USA). Antibodies against AKT, phospho-MEK, MEK, and Cyclin D1 were purchased from Cell Signaling Technology and used as primary antibodies for immunoblotting.

### 4.4. Cell Viability Assay

Cell viability was assessed using the resazurin method [ready-to-use solution; Tokyo Chemical Industry, Tokyo, Japan]. Briefly, cells were seeded in 96-well plates at a density of 5 × 10^3^ cells per well and allowed to adhere overnight. Cells were treated with various concentrations of acyclic retinoids with/without dabrafenib and trametinib for 72 h, and cells treated with an equivalent volume of dimethyl sulfoxide (vehicle) served as the negative control. After treatment, ready-to-use resazurin solution was added at a volume equal to 10% of the cell culture medium volume, and the cells were incubated for 2 h. Finally, the absorbance of each well was measured at 570 nm using a microplate reader (Model 630; Bio-Rad Laboratories, Hercules, CA, USA). Cell viability was expressed as the percentage of OD of control wells treated with dimethyl sulfoxide (100%).

### 4.5. Colony Formation Assay

A375PDTR-D cells were seeded in 6-well plates at a density of 1.0 × 10^3^ cells/well and cultured for 10 days in medium containing vehicle or ACR, with ACR maintained throughout the assay until visible colonies formed. Colonies were fixed, stained, and counted as colonies containing ≥ 50 cells. Data are presented as mean ± SD (*n* = 4). The experiment was conducted in accordance with the standardized in vitro clonogenic assay guidelines [55].

### 4.6. Immunoblotting

Cells treated with or without reagents were washed twice with ice-cold phosphate-buffered saline and lysed in 100 μL of lysis buffer [20 mM HEPES (pH 7.4), 150 mM NaCl, 12.5 mM β-glycerophosphate, 1.5 mM MgCl2, 2 mM Ethylene Glycol Bis(beta-aminoethylether)-N,N,N,N-tetraacetic acid (EGTA), 10 mM NaF, 2 mM dithiothreitol, 1 mM Na3VO4, 1 mM phenylmethylsulfonyl fluoride, 20 µg/mL aprotinin, and 0.5% Triton X-100]. The inhibitor-containing lysis buffer was prepared immediately before use; labile protease/phosphatase inhibitors, including sodium orthovanadate, phenylmethylsulfonyl fluoride, and aprotinin, were added fresh at the time of lysis. Whole cell lysates were resolved by sodium dodecyl sulfate–polyacrylamide gel electrophoresis and transferred to a ClearTrans PVDF membrane (FUJIFILM Wako Chemicals). The membranes were immunoblotted with antibodies described earlier, and the bound antibodies were visualized with horseradish peroxidase-conjugated antibodies against rabbit or mouse IgG (Calbiochem, Darmstadt, Germany) using ImmunoStar Zeta or ImmunoStar LD (FUJIFILM Wako Chemicals). Images were captured using a Lumicube (Liponics, Tokyo, Japan). The bands were quantified using Just TLC software (version 4.7, Liponics, Tokyo, Japan). Protein expression was normalized to that of α-tubulin.

### 4.7. Statistical Analysis

All experiments were performed independently at least thrice. Data are presented as mean ± standard deviation (SD). Statistical analyses were performed using JMP Pro (version 16.0.0; SAS Institute, Cary, NC, USA). Prior to the analyses, the normality of the data distribution was assessed using the Shapiro–Wilk test, and the homogeneity of variance was tested using Levene’s test. Multiple group comparisons were conducted using one-way analysis of variance, followed by Tukey−Kramer post hoc tests. Statistical significance was set at *p* < 0.05.

## 5. Conclusions

In A375-derived dabrafenib plus trametinib-resistant melanoma cells, ACR reduced short-term viability and clonogenic growth in vitro and was associated with reduced STAT3 Tyr705 phosphorylation, lower EGFR, Cyclin D1, and Cyclin B1 expression, and increased p27KIP1. Under the tested conditions, ACR did not detectably suppress AKT, MEK/ERK, or basal JNK phosphorylation. These data support further evaluation of ACR as a candidate non-MAPK adjunct strategy, but the present study does not establish pathway causality, formal synergy, broad model generalizability, clinically achievable exposure, or in vivo efficacy.

## Data Availability

The data presented in this study are available on request from the corresponding author due to their being part of an ongoing study.

## Supplementary Materials

The following supporting information can be downloaded at https://www.mdpi.com/article/10.3390/ijms27146245/s1.
