# Supplementary material for: Acyclic Retinoid Attenuates STAT3 Signaling and Reduces In Vitro Growth of A375-Derived Dabrafenib Plus Trametinib-Resistant Melanoma Cells
Source: Int J Mol Sci. 2026 Jul 14;27(14):6245. doi: 10.3390/ijms27146245 (PMC13412076; doi:10.3390/ijms27146245)
Supplement: Supplementary file 1 [file ijms-27-06245-s001.zip › ijms-4342922-supplementary.pdf]

Supplementary information

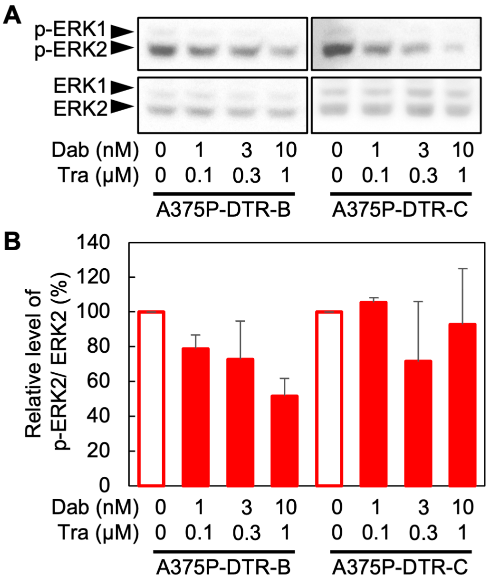

Supplementary Figure S1: The following supporting information can be made available online: Figure S1. Changes in ERK2 phosphorylation levels upon exposure to Dab + Tra in A375PDTR-B and -C. A: Photograph of a western blot showing p-ERK1/ERK1 and p-ERK2/ERK2. B: The relative amounts of p-ERK2/ERK2 were quantified and normalized to the vehicle = 100% for each cell line. Bars indicate mean ± SD (n = 3). Statistical analysis was performed using one-way ANOVA (Tukey–Kramer), and no significant differences from the vehicle control were detected within each cell line.
